# Intrapartum Subarachnoid Migration of Epidural Catheter Inserted for Labour Analgesia: A Case Report

**DOI:** 10.7759/cureus.36979

**Published:** 2023-03-31

**Authors:** Priya V Sambhukumar, Riaz Mohamed, Anagha Shankar

**Affiliations:** 1 Anesthesiology, NMC Specialty Hospital, Abu Dhabi, ARE; 2 Anatomical Sciences, Humanitas University, Milan, ITA

**Keywords:** intrathecal migration, subarachnoid migration, labour analgesia, intrapartum, epidural catheter

## Abstract

Epidural analgesia is commonly used for pain management during labor. Owing to the blind nature of the insertion of the catheters, they are prone to migration to various spaces intra-spinally, which may result in a multitude of complications. We present a case of a 32-year-old lady who was admitted with labor pain, and an epidural catheter was inserted for labor analgesia. Five hours after insertion, she developed sudden motor and sensory impairment suggestive of subarachnoid migration of the catheter. The diagnosis, management, and risks associated with delay in the identification of this potentially fatal complication are discussed.

## Introduction

Epidural analgesia is a very popular and effective modality used to alleviate pain during labor. Although it is widely used in obstetric practice, complications are not uncommon. Injury to the epidural veins during insertion can cause hematomas, and the catheter may also enter the veins, resulting in systemic toxicity due to the local anesthetic drugs. The catheter can also migrate inwards into the subdural or subarachnoid space or outwards, out of the spinal canal. Unintentional subarachnoid injection of large volumes of local anesthetic can lead to high or total spinal anesthesia. Our case describes a delayed intrathecal migration and high spinal anesthesia from a previously functioning epidural catheter.

## Case presentation

A 32-year-old lady, G2P1L1 (Gravida 2, Para 1, Live 1) with a history of previous caesarean section was admitted with labor pain and was planned for vaginal delivery by the obstetrician. She had a pain score of 6/10 and was offered epidural analgesia. The procedure and the possible complications were discussed and informed consent was taken. The patient was placed in the right lateral position and the L3-4 interspace was selected. An epidural needle (18 gauge, Portex-Minipack System I^®^) was inserted through the midline after cleaning and draping the area. Epidural space was identified by the loss of resistance to air at 6cm from the skin. After the aspiration was negative for blood or cerebrospinal fluid, a test dose of 3ml of 0.5% ropivacaine (1.5ml of 1% ropivacaine diluted with 1.5ml of saline) was given, which did not cause any motor block, confirming that the needle was not in the subarachnoid space. The epidural catheter was then introduced and fixed with the 11cm marking at the skin level. A bolus dose of 5ml of 0.2% ropivacaine with 25 micrograms of fentanyl was given. The patient was then turned to the supine position and monitoring of vital signs and cardiotocograph was continued.

After 10 minutes the pain score was zero. Sensory level was at D6 to D8 level and there was no motor block. She was advised to alternate between the left and right lateral position every 30 to 45 minutes. One hour later, continuous infusion of 0.2% ropivacaine with fentanyl 1microgram/ml was started at a rate of 5ml per hour. She was comfortable and her vitals were stable on this regimen. Four hours after initiation of the infusion, that is, five hours after insertion of the catheter, she complained of itching over the anterior chest and loss of sensations over the trunk and lower limbs. She was also unable to move her lower limbs. She was conscious, oriented and her vitals were stable. On examination, she was found to have sensory loss from below the level of the clavicles bilaterally with grade zero power in the lower limbs. The epidural infusion was stopped immediately. The position of the epidural catheter was checked to confirm that it had not migrated inwards or outwards after the insertion. Once this was ascertained, aspiration from the epidural catheter was attempted, and a free flow of clear fluid was seen. Laboratory examination of this fluid for glucose confirmed that it was cerebrospinal fluid.

**Table 1:** Fluid analysis confirming intrathecal migration of catheter

CSF Test	Results	Reference range
Cell count - WBC	0 cells/mm^3^	0-5cells/mm^3^
Cell count - RBC	<5 cells/mm^3^	Absent
Glucose	4.94mmol/L	2.22-3.88mmol/L
Protein	54.97mg/dL	15-45mg/dL

The patient was reassured and the fact that the catheter had probably migrated intra-thecally was explained to her and her spouse. She was monitored closely and equipment for emergency intubation was kept by the patient’s bedside, as was a crash cart. Obstetric evaluation at this time showed that the cervix was dilated to 8cm. Artificial rupture of the membrane was done and Pitocin® (oxytocin) infusion was started as per the protocol by the obstetrician. One hour later the sensory level had regressed to the D5-6 level. Another hour later, her sensory level was at D10 and her cervix was fully dilated. As the power was still grade zero, she was delivered with Kiwi cup vacuum assistance. After the delivery, the placenta and membranes were expelled completely and there was no post-partum hemorrhage. The uterus had contracted well. Four hours after delivery her sensations recovered completely and she also started to move her legs. The intrathecal catheter was left in situ for 24 hours and then removed. She was then ambulated and was initially comfortable, but developed an occipital headache about 12 hours later. The pain was more in the sitting position. The possibility of post dural puncture headache and the different modalities for management of the same was explained to her. She was initially managed with non-steroidal anti-inflammatory drugs (NSAID) but the next day her headache worsened and was associated with neck pain and tinnitus in the right ear. As she did not wish to undergo an epidural blood patch, she was managed with paracetamol, caffeine, NSAID, bed rest and parenteral fluids. Over the next 48 hours, her symptoms gradually subsided and she was discharged from the hospital.

## Discussion

The tendency of epidural catheters to move away from their intended site of placement has been common knowledge for decades. Day and Graham noted in 2002 that this happens in up to 50% of cases [1]. A study by Lim et al. found that when the catheter has to be threaded to a location remote from the site of entry, only 13% could be threaded for over 4cm from the needle tip without coiling [2]. Similarly, only 35% remained in the posterior epidural space when threaded beyond 2cm [3]. This tendency to wander results in the catheter tip winding up within the subdural or subarachnoid spaces, inside blood vessels (usually through the epidural venous plexus) as well as extra-spinally in the muscular or subcutaneous planes [4-6]. In most cases, the migration of the catheter is discovered immediately after insertion, either when blood or cerebro-spinal fluid is aspirated through the catheter, or when the patient develops a neuromuscular blockade on administration of a test dose of local anesthetic medication. The incidence of an apparently normally functioning epidural catheter moving away from its initial position and resulting in a neurological deficit hours or days after it was initially inserted is much lower and only a few cases have been reported in literature since the first such case, which was reported in 1985 [7-10].

The mechanism behind such apparently spontaneous penetration of the dura mater by the catheter tip has been a matter of detailed study. Post mortem evaluations of the dura mater showed that it was relatively resistant to puncture by the catheters and hence most authors have hypothesised that a weakness in the dura, probably secondary to an injury during the insertion of the Tuohy needle, is a perquisite for delayed migration of the catheters subdurally. If the catheter is positioned with its tip in the subdural space and the side holes in the epidural compartment, the relative negative pressure in the epidural space may lead to preferential drainage of the infusion to this compartment. Sudden rupture of the arachnoid while administering bolus doses or its rupture following gradual build-up of pressure in the subdural space over a period of time as the infusate collects in this space has been postulated as a cause for the delayed presentation of migrated catheter tips [4,11,12].

The thickness of the spinal dura progressively reduces from about 2.5mm in the cervical region to 0.5mm in the lumbo-sacral area, and this may be a contributory factor in increased incidence of accidental dural punctures in the lumbar region [13]. The increased movements of parturient women as the labor progresses may also be a contributory factor [14].

High regional block is an inadvertent complication of neuraxial anesthesia. This has been arbitrarily classified into high spinal when the local anesthesia spreads above the D4 spinal level and total spinal when the block extends intracranially. High spinal anesthesia results in the blockade of the cardiac sympathetic fibers causing hypotension and bradycardia. Extension to the cervical spine presents as numbness and weakness of hands/grip when C6-8 levels are affected. Involvement of C3-5 levels may result in diaphragmatic paralysis manifesting as breathing difficulty or respiratory failure. Total spinal block causes slurring of speech followed by loss of consciousness and cardio-respiratory arrest [15].

Initial management consists of immediate cessation of infusion of the anesthetic medication, administration of oxygen and repositioning the patient in a reverse Trendelenburg position with uterine displacement to the left. First, reassure the patient and relatives. Next, keep resuscitation equipment and emergency medications ready to tackle any further deterioration in the patient’s condition. Hypotension caused by a high spinal block requires intravenous fluids and vasopressors like ephedrine or phenylephrine. Total spinal block requires emergency rapid sequence intubation and ventilation till the neurological function recovers. In case of cardiac arrest, cardiopulmonary resuscitation is started and an emergency caesarean section has to be done within four minutes to rescue the baby [15].

Early detection of a high neuraxial block depends on close monitoring of the patient. Other than the vital signs, the sensory and motor level should be monitored. Constant communication with the patient alleviates anxiety and also helps to detect speech changes and breathing difficulties early.

Once an intrathecal position of the catheter is confirmed, it should be left in situ as it can be used to administer spinal anesthesia if the patient needs to undergo a caesarean section. Additionally, studies have shown that this reduces the incidence of post dural puncture headaches [16]. In our patient, the intrathecal catheter was left in situ for a period of 24 hours after the discovery of the accidental catheter migration. However, she still developed a headache which was resolved with conservative management.

## Conclusions

Intrapartum epidural analgesia is an integral part of modern obstetric practice. The pain management team must be aware of the rare but potentially lethal complication of high or total spinal anesthesia resulting from intrathecal migration of the epidural catheter. Since early detection is the key to management, continuous close monitoring of all patients who are receiving epidural analgesia must be emphasized to all caregivers. Adequate training, awareness of the potential for catheter tip migration and availability of essential drugs and life-saving equipment will help to manage this rare complication successfully.
